# Performance of new pp65-IGRA for the quantification of HCMV-specific CD4^+^ T-cell response in healthy subjects and in solid organ transplant recipients

**DOI:** 10.3389/fimmu.2025.1553305

**Published:** 2025-05-15

**Authors:** Dalila Mele, Federica Zavaglio, Federica Bergami, Marilena Gregorini, Domenica Federica Briganti, Carlo Pellegrini, Giuditta Comolli, Irene Cassaniti, Daniele Lilleri, Fausto Baldanti

**Affiliations:** ^1^ Molecular Virology Unit, Department of Microbiology and Virology, Fondazione IRCCS Policlinico San Matteo, Pavia, Italy; ^2^ Unit of Nephrology, Dialysis and Transplantation, Fondazione IRCCS Policlinico San Matteo, Pavia, Italy; ^3^ UOS Transplant Center, Fondazione IRCCS Policlinico San Matteo, Pavia, Italy; ^4^ Department of Clinical, Surgical, Diagnostic and Pediatric Sciences, University of Pavia, Pavia, Italy; ^5^ Department of Cardiothoracic Surgery, Fondazione IRCCS Policlinico San Matteo, Pavia, Italy

**Keywords:** HCMV, IFN-γ, IGRA, pp65, HCMV-specific T cell response

## Abstract

Immune control of human cytomegalovirus (HCMV) replication is critical in bone marrow and solid organ transplant recipients, where uncontrolled replication can lead to high mortality. Current commercial immune monitoring tools have several limitations, such as a lack of appropriate test cutoff values and the inability to characterise antigen-specific T cells. The main aim of our study was to develop a new interferon-γ (IFN-γ) release assay (IGRA), easy to use, to quantify and characterise the HCMV-specific T-cell response (pp65-IGRA). Secondary analyses included an evaluation of the performance of pp65-IGRA to assess whether its specificity and sensitivity were equal to or greater than those of the intracellular cytokine staining (ICS) and enzyme-linked immunospot (ELISpot) assays. In the study, 76 immunocompetent donors and nine solid organ transplant recipients were enrolled. Blood samples or peripheral blood mononuclear cells were stimulated with HCMV pp65-recombinant protein or with a complete pool of overlapping pp65 peptides. IFN-γ production was analysed by enzyme-linked immunoassay, ELISpot assays, and flow cytometry. For each assay, appropriate cutoff values were calculated. Our data demonstrate the suitability of pp65-IGRA for the quantification of HCMV-specific CD4^+^ T-cell responses and may support its use in routine clinical practice to improve the management of immunocompromised patients.

## 1 Introduction

Human cytomegalovirus (HCMV) is a ubiquitous DNA virus capable of establishing lifelong latency in bone marrow hematopoietic progenitor cells after primary infection (1). Periodically, a latently infected virus can restart replication, causing reactivation episodes.

Upon primary HCMV infection, the virus can trigger an overwhelming response involving many arms of the immune system (2). Several studies have documented that cell-mediated adaptive immunity (CMI) plays a key role in the control of the replication of HCMV (3, 4). Particularly, HCMV-specific CD8^+^ T lymphocytes are essential for limiting HCMV viremia during the acute phase of primary infection, whereas long-term immune control of infection is established by the CD4^+^ T lymphocyte subset. Indeed, several works, including ours, give direct evidence that the presence of an HCMV-specific CD4^+^ T-cell response is associated with a lower risk of HCMV disease (5–8). HCMV infection or reactivation in the immunocompetent individual is rarely a cause of morbidity. Conversely, the reduced immune response in bone marrow or solid organ transplant recipients, due to immunosuppressive therapies, makes them susceptible to viral reactivation with serious life‐threatening risks (3).

Current guidelines suggest two main strategies to prevent HCMV disease in transplant recipients: the universal prophylaxis (based on administration of antiviral drugs to all patients for up to 12 months) and preemptive therapy (based on monitoring the viral burden in the blood and treatment when transplant recipients are deemed to be at high risk (9, 10). Both approaches have limitations, such as cost, toxicity, and risk for emergence of resistance. However, patients without significant HCMV-specific T-cell dysfunction could avoid both prophylactic and preventive therapies. In fact, HCMV reactivation episodes and the risk of disease are associated with each patient’s immune status, and transplant recipients who maintain a sufficient HCMV-specific T-cell response can control HCMV infection despite immune suppression (5, 8, 11–15). Therefore, it is important in the clinical practice the employment of HCMV–CMI assays, particularly those that track the specific CD4 T-cell response, to guide personalized strategies aimed at preventing HCMV in immunocompromised individuals (11–15).

Different clinical tools have been evaluated for *ex-vivo* quantitation and functional characterization of antigen-specific T-cell responses, including enzyme-linked immunospot (ELISPOT), enzyme-linked immunoassay (ELISA), and flow cytometry. Of these, ELISA and ELISpot are highly specific and sensitive but do not provide the phenotypic characterization of antigen-stimulated T cells (16–18). On the other hand, the flow cytometry approach allows the analysis of cell function and phenotype in parallel (5, 8, 14, 15), but it is labor intensive, expensive, and poorly standardized. Moreover, flow cytometry or ELISpot requires trained operators to perform the tests accurately and interpret the results. The preparation of peripheral blood mononuclear cells (PBMCs) requires considerable expertise. Specifically, PBMCs should be used or cryopreserved within hours of blood collection to ensure data quality. Therefore, whole blood assays could be more advantageous than PBMC-based methods by significantly reducing blood volume, being rapid and automated, and not requiring skilled personnel. The QuantiFERON-CMV assay is the only commercially available method for measuring CMI response in whole blood samples. It is an *in-vitro* assay using HCMV peptides that are designed to specifically target CD8+ T cells and are restricted by HLA class I haplotypes, which cover > 98% of the human population. Therefore, this test is not suitable for subjects with HLA class I haplotypes that are not covered (18, 19). Additionally, it does not analyze HCMV-specific CD4+ T-cell responses. Several studies have reported that 15- to 20-mer overlapping peptides are able to stimulate both CD4^+^ and CD8^+^ T cell immunity, whereas whole proteins mainly stimulate CD4^+^ T cells (20, 21). The aim of our study was to develop a novel, easy-to-perform, whole-blood Interferon-Gamma-Release Assay (IGRA) that requires minimal blood volume and is suitable for accurate quantification of HCMV-specific CD4^+^ T-cell response and to compare its performance with that of the currently available assays. For this reason, whole blood samples were stimulated with HCMV pp65-recombinant protein or a complete pool of overlapping pp65 peptides (pp65-IGRA). Additionally, three different HCMV-specific IGRAs were evaluated and compared with the novel pp65-IGRA: intracellular cytokine staining (ICS) by flow cytometry, ELISpot assay developed in our institute, and HCMV–IFN-γ ELISA (QuantiFERON-CMV, Germany, Qiagen). Of note, the ELISpot assay detects overall specific T-cell response, whereas the QuantiFERON-CMV assay measures HCMV-CMI by quantifying IFNγ released by CD8^+^ T cells.

## 2 Materials and methods

### 2.1 Study setting

For the setup and the comparative evaluation of pp65-IGRA, peripheral blood samples were collected from 76 immunocompetent donors. In addition, blood samples were collected from 9 HCMV-seropositive solid organ transplant recipients (SOTR) before and 3 months after transplantation to test preliminarily pp65-IGRA in this population. PBMCs were obtained from heparin-treated blood by density gradient centrifugation (Lymphoprep, Sentinel Diagnostics, Milan, Italy) and were used to measure antigen-specific T-cell responses by ICS and ELISpot assay. Serum samples were used for HCMV IgG serology. All subjects signed an informed consent form. The study was approved by the local Ethics Committee (Comitato Etico Area Pavia) and institutional review board (Prot. 0003690/2024).

### 2.2 HCMV serology

For quantifications of anti-HCMV IgG antibody titre in serum, the automated chemiluminescence analyser technology was used (LIASON XL, Italy, DiaSorin). Values lower than 12 mUI/ml were considered negative.

### 2.3 Media and antigens

To evaluate the HCMV-specific T-cell response, recombinant pp65 protein (Abcam, Cambridge, UK) and pp65 peptide pool (15 mers, overlapping by 10 amino acids, A&A Labs LLC, San Diego, CA) were used at a final concentration of 1 µg/ml. Commercial phytohaemagglutinin (PHA, 5 μg/ml; MO, USA) or SEB (Staphylococcus aureus, Enterotoxin Type B, 10 µg/ml) was used as a positive control in the ELISpot assay and pp65-IGRA whole blood assay. A peptide pool of human actin (15 mers, overlapping by 10 amino acids, Pepscan, Lelystad, the Netherlands) was used as a negative control in the ICS assay at a final concentration of 1 µg/ml. Culture medium was RPMI 1640 (Euroclone, Milano, Italy) supplemented with 2 mM L-glutamine (Euroclone), 100 U/ml penicillin and 100 µg/ml streptomycin solution (Euroclone), and 10% of heat-inactivated fetal bovine serum.

### 2.4 Intracellular cytokine staining assay

In a round-bottom 96-well plate, peripheral blood mononuclear cells (PBMCs) were stimulated for 16h–18h (22, 23) with recombinant pp65 protein, pp65 peptide pool, and peptide pool of human actin in the presence of 0.5 µg/ml co-stimulator molecules, CD28 and CD49d (BD Bioscience, New Jersey, USA), and brefeldin A (Sigma-Aldrich-Merck, Darmstadt, Germany) at a final concentration of 10 µg/ml. Cells were seeded at a density of 0.5–1 × 10^6^ cells/200 µl culture medium per well. Cells were then incubated overnight at 37°C (5% CO_2_). Subsequently, PBMCs were harvested, washed, and stained using CD8 V500, CD3 PerCP-Cy 5.5, and CD4 APC Cy7 (BD Biosciences). After fixation and permeabilization (Fixation/Permeabilization Solution Kit, BD Biosciences), cells were stained with anti–IFN-γ PECy7 (BD Biosciences). Nonviable cells were identified by staining with Live/Dead Fixable Violet Dye Pacific Blue (Invitrogen, MA, USA). Data acquisition was performed with a FACS Lyric flow cytometer using BD FACSuite software (BD Biosciences) (23–25). The frequency of IFN-γ–producing CD4^+^ and CD8^+^ T cells is determined by subtracting the frequency of IFNγ^+^ CD4^+^ or CD8^+^ T cells incubated with human actin peptides from the IFNγ^+^ CD4^+^ or CD8^+^ T cells incubated with recombinant pp65 protein and pp65 peptide pool.

### 2.5 *Ex-vivo* enzyme-linked immunospot assay

Antigen-specific T-cell responses were evaluated by IFN-γ detection following recombinant pp65 protein and pp65 peptide pool stimulation in an ELISpot assay as previously described (26). Negative control wells lacked peptides, and positive control wells contained PHA. Spots were counted using an automated ELISpot Reader System (Autoimmun Diagnostika GmbH, Strasburg, Germany). Results were expressed as IFN-γ spot-forming units (SFUs)/10^6^ PBMCs, after subtracting spots from the negative control.

### 2.6 HCMV-specific interferon-gamma-release assays (pp65-IGRA)

In a 48-well plate, 400 µl of heparinized whole blood were stimulated with the same stimuli used for the ICS assay and maintained overnight at 37°C (5% CO_2_). Unstimulated whole blood was used as a negative control. Subsequently, plasma was harvested and analyzed for IFN-γ [µg/ml ELISA assay, according to manufacturer’s instructions (Quantikine ELISA, R&D Systems, MN, USA)]. The IFN-γ levels of the negative control were subtracted from the unstimulated one.

### 2.7 QuantiFERON-CMV assay

The CE-IVD QuantiFERON-CMV assay had been performed according to the manufacturer’s instructions (Qiagen, Germany). Plasma was harvested and analyzed for IFN-γ (IU/ml) using the QuantiFERON-CMV ELISA kit (Qiagen).

### 2.8 Statistical analysis

GraphPad Prism 9.1.0 (GraphPad Software, La Jolla, CA, USA) was used for statistical analyses. A two-sided *p*-value < 0.05 was considered statistically significant. Receiver operating characteristic (ROC) curve analysis was done to evaluate the optimum cutoffs to discriminate HCMV seropositive and seronegative subjects. The cutoffs were established according to the Youden’s index (or Youden’s *J* statistic) (27), defined as:


J=sensitivity+specificity−1

The maximum value of the index was used as a criterion for selecting the optimum cutoff value, in order to obtain the best compromise between sensitivity and specificity. The area under the curve (AUC) and its 95% confidence interval (CI) were calculated. Correlations between variables were analysed by Pearson’s rank correlation coefficient.

## 3 Results

### 3.1 Demographic characteristics of subjects included in the study

For the evaluation of the efficacy of pp65-IGRA in detecting HCMV-specific T-cell response in immunocompetent subjects and for the comparison of its diagnostic efficacy with that of other assays, we tested 76 immunocompetent donors (48 females and 28 males) whose median age was 50 years (range: 25–89 years).

Detection of HCMV-specific T-cell response by pp65 IGRA was subsequently evaluated in nine SOTR (four females and five males) whose median age was 58 years (range: 19–69 years).

### 3.2 T-cell response to pp65 after incubation of whole blood and PBMCs with a peptide pool or the recombinant protein

**Figure 1:**
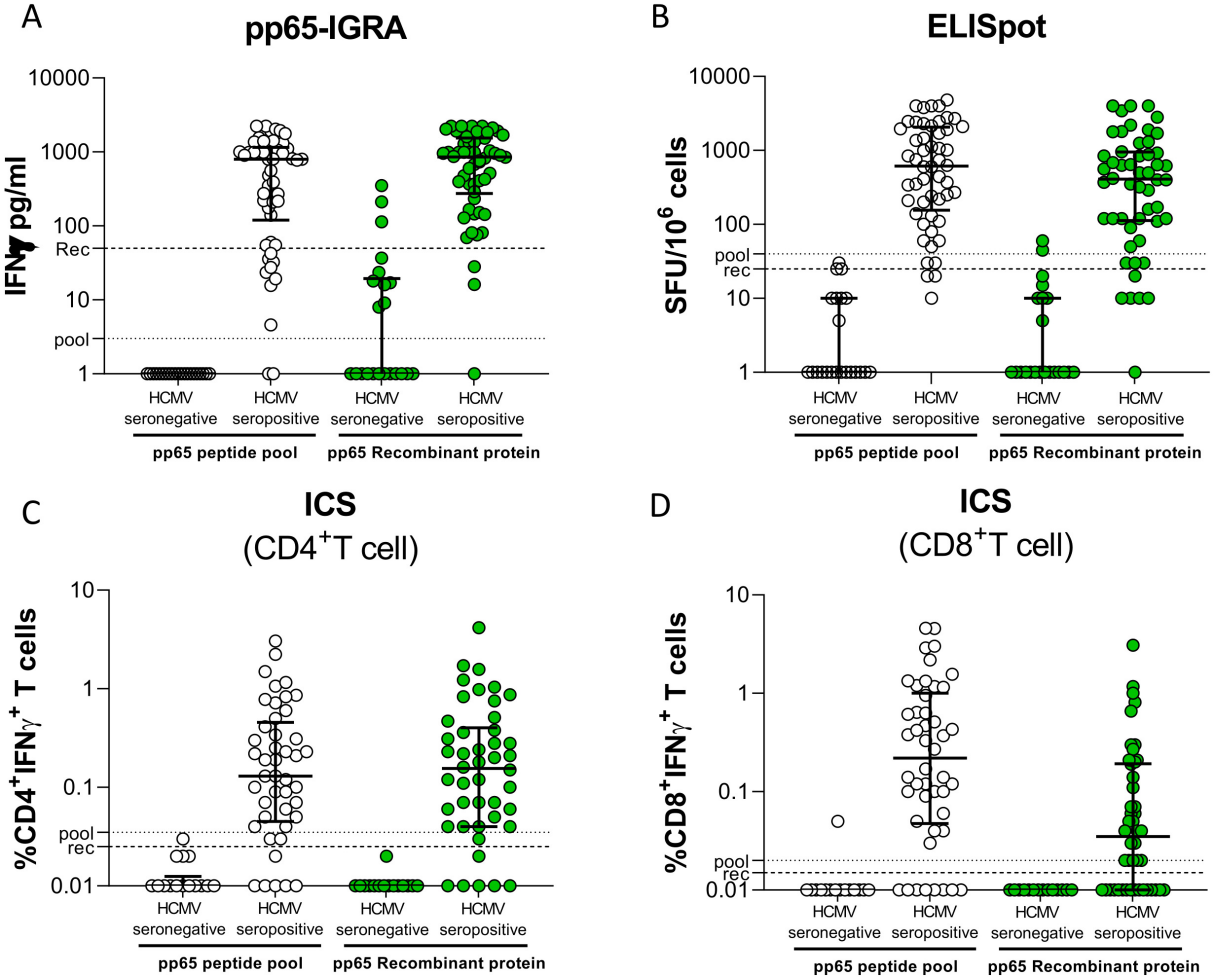
Whole blood IFN-γ release was measured in 22 HCMV-seronegative and 54 HCV-seropositive donors following stimulation with pp65 peptide pool (white dot) or recombinant protein (green dot) **(A)**. Number of spot-forming cells in response to stimulation with pp65 peptide pool or recombinant in stratified HD **(B)**. Frequency of CD4^+^ and CD8^+^ T cells producing IFN-γ in PBMCs of donors stimulated with pp65 peptide pool or recombinant protein (**C**, **D**, respectively). The horizontal dotted line indicated the cutoff.

Whole blood (WB) from 54 seropositive and 22 seronegative immunocompetent donors was incubated with a peptide pool of pp65 or the recombinant protein, and the concentration of IFN-γ released was measured (pp65-IGRA; **Figure 1A**). As expected, both antigen formulations were able to induce IFN-γ release from most seropositive subjects. On the contrary, WB from HCMV-seronegative subjects stimulated with the peptide pool gave a negligible response, while a certain amount of IFN-γ release was observed in a minor portion of recombinant pp65-stimulated WB samples. By ROC analysis, and according to Youden’s index, a cutoff of 3 pg/ml for the peptide pool and a cutoff of 50 pg/ml for the recombinant protein were selected for discrimination of seropositive and seronegative subjects. An ELISpot assay was performed with PBMCs using the same pp65 formulations (**Figure 1B**). Again, IFN-γ–positive spots were produced by the great majority of seropositive subjects, while a small number of spots were produced by few seronegative subjects (the great majority gave negative results). Cutoffs of 40 and 25 SFU/10^6^ cells were chosen for the peptide pool or the recombinant protein. According to the selected cutoffs, after stimulation with the peptide pool, no seronegative subjects gave non-specific results with pp65-IGRA and ELISpot, while using recombinant protein 3 and 2 seronegative subjects gave false-positive results with pp65-IGRA and ELISpot.

IFN-γ production was also determined on CD4^+^ (**Figure 1C**) and CD8^+^ (**Figure 1D**) T cells by ICS. Flow cytometry gate strategies were shown in **Supplementary Figure S1**. As expected, the peptide pool stimulated both T-cell subpopulations, while the recombinant protein provided an excellent stimulation for CD4^+^ and a poor stimulation for CD8^+^ T cells. No seronegative subject gave false-positive results with either peptide pool or recombinant protein in CD4^+^ T-cell response, and one subject gave a false positive result in CD8^+^ T-cell response to peptide pool.

The diagnostic performance of pp65-IGRA, ELISpot, and ICS in discriminating seropositive and seronegative subjects is shown in **Table 1**. A slightly better sensitivity was observed for the pp65-IGRA than the ELISpot. For these two assays, 100% specificity was observed with the peptide pool as stimulus, whereas specificity was close to 100% with the recombinant protein. The ICS assay for CD4^+^ T cells was highly specific with both antigen formulations, while sensitivity was lower than that of pp65-IGRA and ELISpot. The sensitivity was very poor with ICS for CD8^+^ T cells using recombinant protein as stimulus.

**Table 1:** Performance characteristics of all IGRA quantitative assays.

ASSAY	AUC [95% CI]	CUTOFF	Specificity (%)	Sensitivity (%)
pp65-IGRA pp65 pool	0.982 [0.974–1]	3 (pg/ml)	100	96.3
pp65-IGRA pp65 recombinant	0.957 [0.918–0.99]	50 (pg/ml)	94.44	86.36
ELISpot pp65 pool	0.989 [0.954–1]	40 (SFU/10^6^ cells)	100	90.38
ELISpot pp65 recombinant	0.959 [0.914–0.99]	25 (SFU/10^6^ cells)	90.91	88.46
ICS-CD4 pp65 pool	0.917 [0.848–0.985]	0.035 (%)	100	80
ICS-CD4 pp65 recombinant	0.931 [0.869–0.993]	0.025 (%)	100	84.78
ICS-CD8 pp65 pool	0.918 [0.854–0.983]	0.02 (%)	94.44	82.61
ICS-CD8 pp65 recombinant	0.864 [0.779–0.95]	0.02 (%)	100	65.22

AUC, area under curve; 95% CI, confidence interval; SFU, spot forming cells.

### 3.3 Correlation of pp65-IGRA with ELISpot and ICS

**Figure 2:**
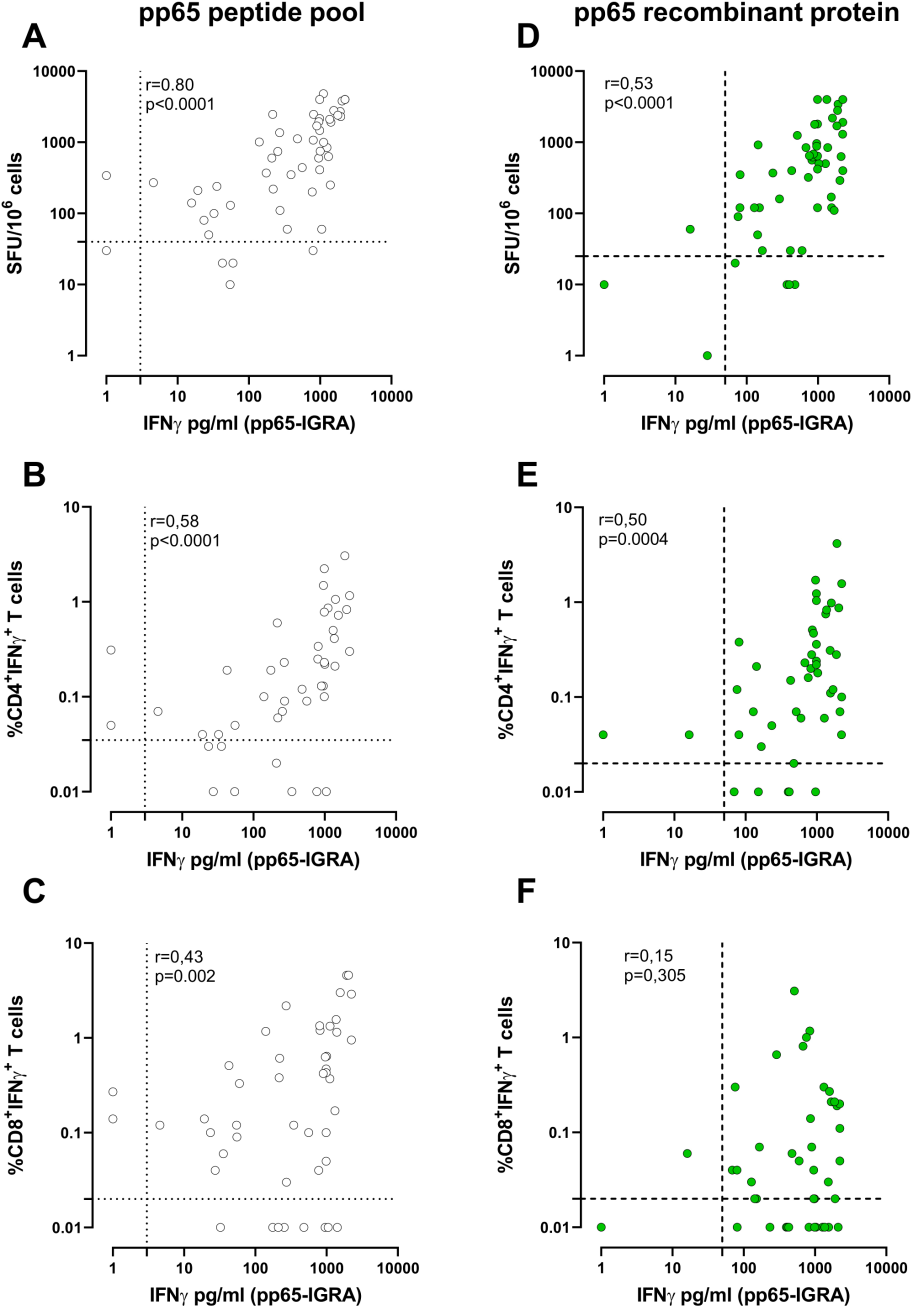
Correlation between whole blood IFN-γ production (pg/ml) and the number of spots on ELISpot following stimulation with pp65 pool (white dots, **A**) or pp65 recombinant (green dots, **D**). Correlation between the IFN-γ production (pg/ml) and the frequency of IFN-γ^+^CD4^+^ **(B, E)** and CD8^+^ T cells **(C, F)** measured by ICS assay following pp65 stimulation. Each dot represents a single sample; Correlation was determined using Spearman, r, and *p*-value are given in the graph. The cutoff line of each analysis was shown.

7Using a peptide pool for T-cell stimulation, among the 54 HCMV-seropositive subjects, there was a significant and high correlation (**Figure 2A**) between pp65-IGRA and ELISpot (*p* < 0.001, *R* = 0.80). A lower correlation (**Figure 2B**) was found between pp65-IGRA and ICS for CD4^+^ T cells (*R* = 0.58), while the lowest correlation (**Figure 2C**) was found between pp65-IGRA and ICS for CD8^+^ T cells (*R* = 0.43). Using the recombinant protein, a significant correlation, albeit low, was found between pp65-IGRA and ELISpot or ICS for CD4^+^ T cells (*R* = 0.53 and 0.51, respectively; **Figures 2D, E**); no significant correlation was observed between pp65-IGRA and ICS for CD8^+^ T cells (**Figure 2F**).

A more complete characterisation of antigen-specific T-cell response can be achieved by using both pp65 formulations. According to the chosen cutoffs, a positive response against both the pp65 peptide pool and the pp65 recombinant protein likely indicates the presence of HCMV-specific CD4^+^ and CD8^+^ T cells. Alternatively, a positive response against the pp65 peptide pool only indicates a response that is primarily associated with CD8^+^ T cells (**Supplementary Figure S2**).

### 3.4 Correlation of QuantiFERON-CMV with pp65-IGRA

**Figure 3:**
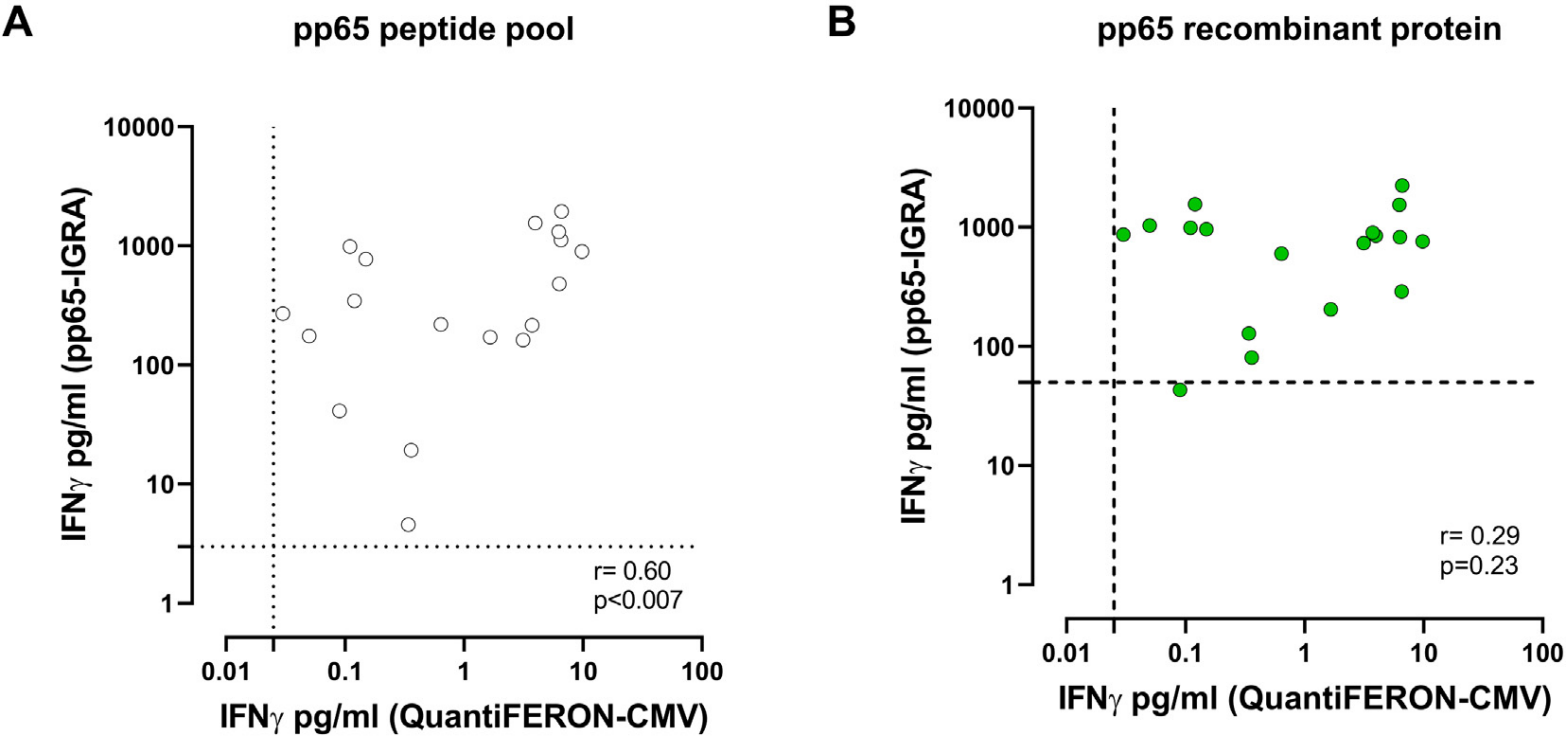
Correlation between IFN-γ level (pg/ml) measured by pp65-IGRA, following stimulation with pp65 pool (white dots, **A**) or pp65 recombinant (green dots, **B**), and IFN-γ level measured by QuantiFERON^®^-CMV. Each dot represents a single sample; correlation was determined using Spearman, r and *p* value are shown in the graph. Cutoff line of each analysis are shown.

In a subgroup of HCMV-seropositive subjects, we analysed the correlation between the commercially available QuantiFERON-CMV, which exploits the incubation of whole blood with CD8^+^ epitopic peptides of known HLA-restriction derived from different HCMV proteins and pp65-specific pp65-IGRA. There was a good correlation between the two assays when the peptide pool of pp65 was used in the pp65-IGRA (*R* = 0.72; **Figure 3A**), while the correlation was lower when the recombinant protein was used (*R* = 0.50; **Figure 3B**).

### 3.5 T-cell response measured by pp65-IGRA in transplant recipients

**Figure 4:**
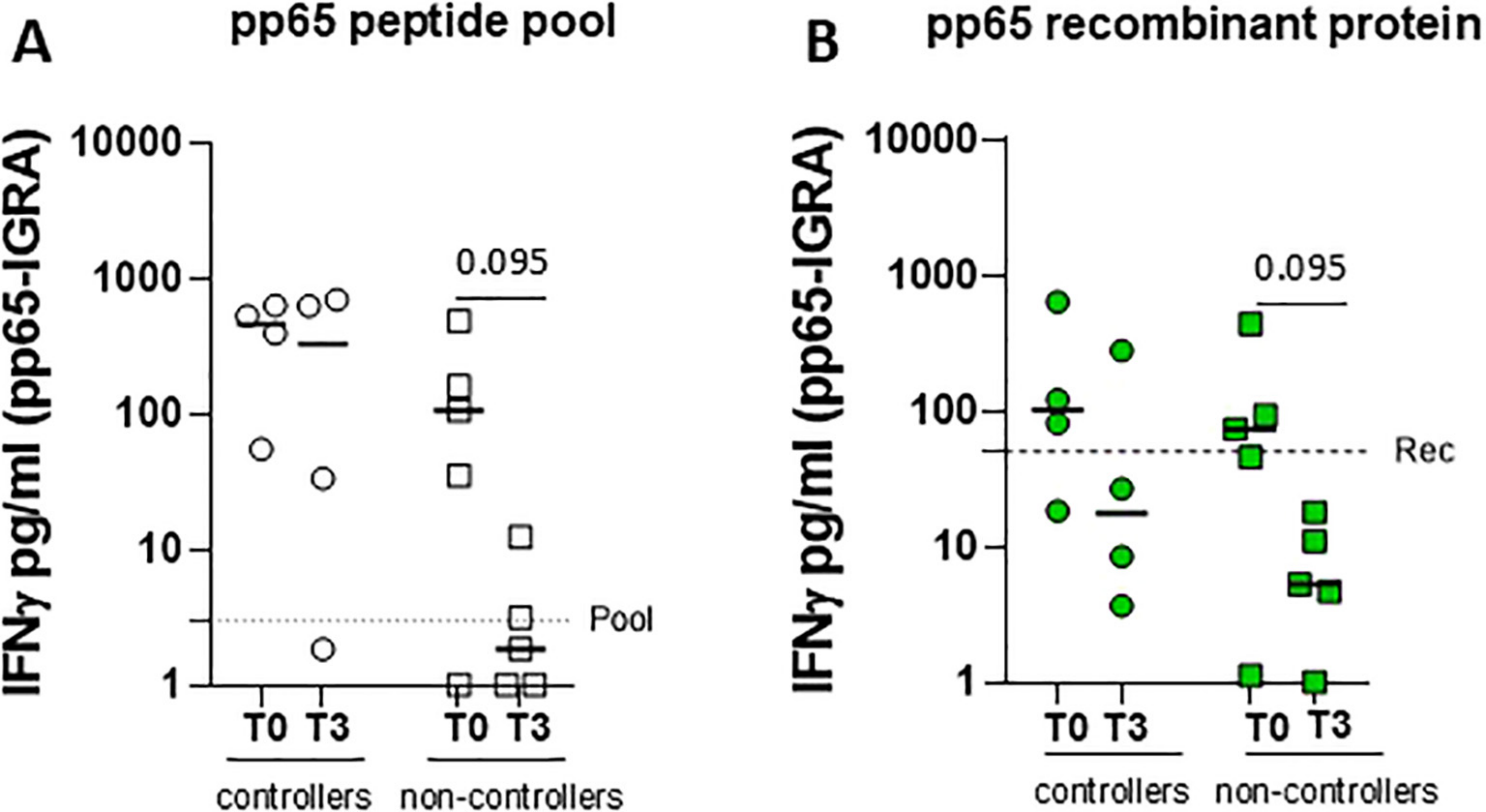
Whole blood IFN-γ release was measured in four controllers (circle dots) and five non-controllers (square dots), solid organ recipients (SOTR) **(A)** before (T0) and 3 months after transplantation. Graphs show levels of IFN-γ released following stimulation with pp65 peptide pool (**A**, white) and recombinant protein (**B**, green) in two groups of SOTR. The horizontal dotted line indicated the cutoff. Statistical analysis was performed by the Mann–Whitney test. *p*-values < 0.1 were shown in the graphs.

Finally, the novel pp65-IGRA assay was used to investigate the antigen-specific T-cell response in nine HCMV-seropositive solid organ recipients before (T0) and 3 months (T3) after transplantation. Overall, 4 of 10 patients were defined as “controllers” due to self-resolving HCMV infection and 5 of 10 patients were defined as “non-controllers” due to needing preemptive therapy. **Supplementary Table S1** shows the clinical and demographic characteristics of two groups of patients. At T0, all SOT recipients except one (eight of nine) were able to induce IFN-γ release after stimulation with a peptide pool of pp65, while seven of nine patients showed a positive pp65-specific T-cell response using the recombinant protein. Comparison of the T-cell response before and after 3 months of transplantation in the two groups of patients showed that “non-controllers” had a reduction in T-cell response as measured by pp65-IGRA, whereas “controllers” maintained higher levels of T-cell response despite immunosuppression. This reduction was more clearly observed, although not statistically significant, when using the pp65 recombinant protein rather than stimulating with the peptide pool (**Figure 4**). Indeed, five of five non-controllers showed a response below the “recombinant protein” cutoff at 3 months after transplantation.

## 4 Discussion

This study reports the evaluation of a new whole blood IGRA for HCMV using pp65 as stimulus. Two different antigenic formulations were used: a pool of overlapping peptides of 15 aa spanning the entire protein and the recombinant whole protein. Data provided by the pp65-IGRA were compared with those provided by an ELISpot assay using the same antigenic formulations, while ICS was also performed to analyze the relevant contribution of CD4^+^ or CD8^+^ T cells in IFN-γ production. Results of the study show that pp65-IGRA is able to detect a T-cell response in the majority of seropositive subjects tested (96%) with either peptide pool or recombinant protein, whereas few seronegative subjects gave non-specific results with the recombinant protein, and no subject gave non-specific results with the peptide pool.

Over the past 2 decades, there has been a push to develop HCMV-specific CMI assays that can accurately measure the HCMV-specific T-cell response, an important predictor of HCMV disease in transplant recipients. Current guidelines endorse the use of HCMV-specific T-cell response monitoring to inform on the risk of HCMV infection (28, 29). ELISpot or QuantiFERON-CMV assays have been widely used for monitoring the reconstitution or *ex-novo* development of HCMV-specific T-cell response in the post-transplant period (30, 31) and to individualize the duration of antiviral prophylaxis (11, 12, 32).

Direct comparison of ELISpot and QuantiFERON (33–35) reported a better performance of ELISPOT in transplant recipients. The commercially available ELISpot and QuantiFERON-CMV assays, although standardised and CE-marked, exhibit drawbacks that hinder their routine use in clinical practice. The former is highly specific and sensitive but does not provide phenotypic characterization of antigen-stimulated T cells. The latter, QuantiFERON-CMV (Qiagen Inc.), is a standardised and easy-to-perform assay based on a stimulation with HLA class 1–restricted HCMV epitopes; therefore detects mainly CD8+ T-cell response and cannot discriminate CD4+ T cells producing IFN-γ.

It is interesting to note that our new assay, pp65-IGRA, showed a slightly better sensitivity than that observed for the ELISpot. We cannot exclude a potential impact of the sample preparation (whole blood vs. PBMCs) on the different performance of the assays.

In addition, the pp65 overlapping peptide pool was shown to stimulate both CD4^+^ and CD8^+^ T-cell responses simultaneously, whereas the whole protein was observed to elicit predominantly HCMV-specific CD4+ T-cell responses, which have been reported to be crucial for immune control of CMV viremia after transplantation (5–8). This is further supported by the weak correlation observed between the pp65-IGRA and QuantiFERON-CMV when recombinant protein was used in the pp65-IGRA. Moreover, the QuantiFERON-CMV assay is limited by the HLA type of the patient; the assay is based on the stimulation of CD8+ T cells with a pool of 22 short peptides from 6 HCMV proteins presented by several HLA class I haplotypes, but we showed that mismatching between patient HLA alleles and those cognate to peptides present in the QuantiFERON-CMV pool may impact on the results obtained (19, 25). Conversely, the pp65-IGRA involves overlapping peptides of the pp65 antigen, therefore being able to detect a T-cell response to pp65 independently from specific patient HLA type.

The production of IFN-γ in response to recombinant pp65 found in certain HCMV-seronegative subjects may depend on protein formulation (e.g., purity level, endotoxin presence). It is also possible that the recombinant protein activates the innate immune response in a non-specific manner, inducing IFN-γ production. In addition to T and NK cells, monocytes and macrophages have also been reported to produce IFN-γ (36, 37). We could speculate that in some subjects the recombinant protein may induce IFN-γ production by monocytes or macrophages through the activation of the TLR2, TLR3, or TLR4 pathway, as usually occurs with other microbial products. These facts may be at the basis of a specificity slightly below 100% for the recombinant protein. On the other hand, we cannot completely exclude a humoral/cellular mismatch in these donors, since subgroups of healthy donors who, despite being HCMV-seronegative, show CD4^+^ and CD8^+^ T-cell responses have been described (38).

Our study is limited to the evaluation of the efficacy of pp65-IGRA mainly in a cohort of immunocompetent individuals stratified by HCMV-serostatus and analyses only a small number of immunocompromised transplanted subjects. Another limitation is the imbalance between male and female donors, which may have influenced the analysis. However, our data demonstrate the suitability of pp65-IGRA for the quantification of HCMV-specific CD4^+^ T-cell responses and its potentiality in identifying patients at risk for, or protected from, HCMV infection after transplantation. As a next step, performance of pp65-IGRA and the cutoff values here determined in immunocompetent subjects should be evaluated on larger cohorts of patients in different transplantation settings (organ or hematopoietic stem cell transplantation, adult or pediatric patients) and receiving different immunosuppressive regimens. Results of these future studies, if confirming the preliminary data presented here, will support the use of the assay in routine clinical practice to improve the management of immunocompromised patients. In particular, results of the assay could be used to identify patients requiring strict HCMV surveillance or antiviral prophylaxis and those who can safely avoid or interrupt anti-HCMV treatment or prophylaxis, therefore improving patient management with a personalized approach to HCMV control, able also to spare costs of unnecessary antiviral drug administration.

## Data Availability

The original contributions presented in the study are included in the article/**Supplementary Material**. Further inquiries can be directed to the corresponding author.
