# Supplementary material for: Performance of new pp65-IGRA for the quantification of HCMV-specific CD4+ T-cell response in healthy subjects and in solid organ transplant recipients
Source: Front Immunol. 2025 May 15;16:1553305. doi: 10.3389/fimmu.2025.1553305 (PMC12119300; doi:10.3389/fimmu.2025.1553305)
Supplement: Supplementary file 1 [file Table1.pdf]

Supplementary Table 1. Clinical and demographic characteristics of patients.

	SOTR Controllers (n=4)	SOTR Non-Controllers (n=5)	*p-Value*
**Age, median** (range)	62 (19-68)	54 (46-69)	>0.999
**Male gender, n (%)**	2	3	-
**CD3 T cells/ul,** median(range) T0	1121 (587-2563)	1088 (674-1763)	>0.999
T3	1514 (527-2563)	317 (177-1775)	0.063
**CD4 T cells/ul,** median(range) T0	652 (321-1286)	739 (472-1415)	>0.999
T3	812 (242-999)	100 (68-905)	0.063
**CD8 T cells/ul,** median(range) T0	418 (227-1286)	311 (198-508)	>0.999
T3	815 (190-1450)	142 (79-232)	0.063
**Transplanted organ**			
Kidney, n	2	5	-
Heart, n	1	0	-
Lung, n	1	0	-
**Immunosuppression Induction**			
No induction, n	2	0	-
MP with basiliximab, n	1	2	-
MP with Thymoglobuline, n	1	3	-
**Immunosuppression Maintenance**			
Tacrolimus with MP, n	2	0	
Tacrolimus with MMF and MP, n	2	5	0.07
**Peak CMV-DNAemia median,** Log(copies/ml) (range)	3.96 (3.29-4.29)	5.7 (5.1-6.38)	***0.015***
**Antiviral therapy**	No	VGCV	–

Statistical analysis was performed by Mann-Whitney U test. p<0.05 was statistically significant. SOTR, Solid organ transplant recipients, T0, before transplantation; T3, 3 months after transplantation; MP, methylprednisolone; MMF, mycophenolate mofetil; VGCV, Valganciclovir
