# Supplementary material for: Performance of new pp65-IGRA for the quantification of HCMV-specific CD4+ T-cell response in healthy subjects and in solid organ transplant recipients
Source: Front Immunol. 2025 May 15;16:1553305. doi: 10.3389/fimmu.2025.1553305 (PMC12119300; doi:10.3389/fimmu.2025.1553305)
Supplement: Supplementary file 2 [file Image1.pdf]

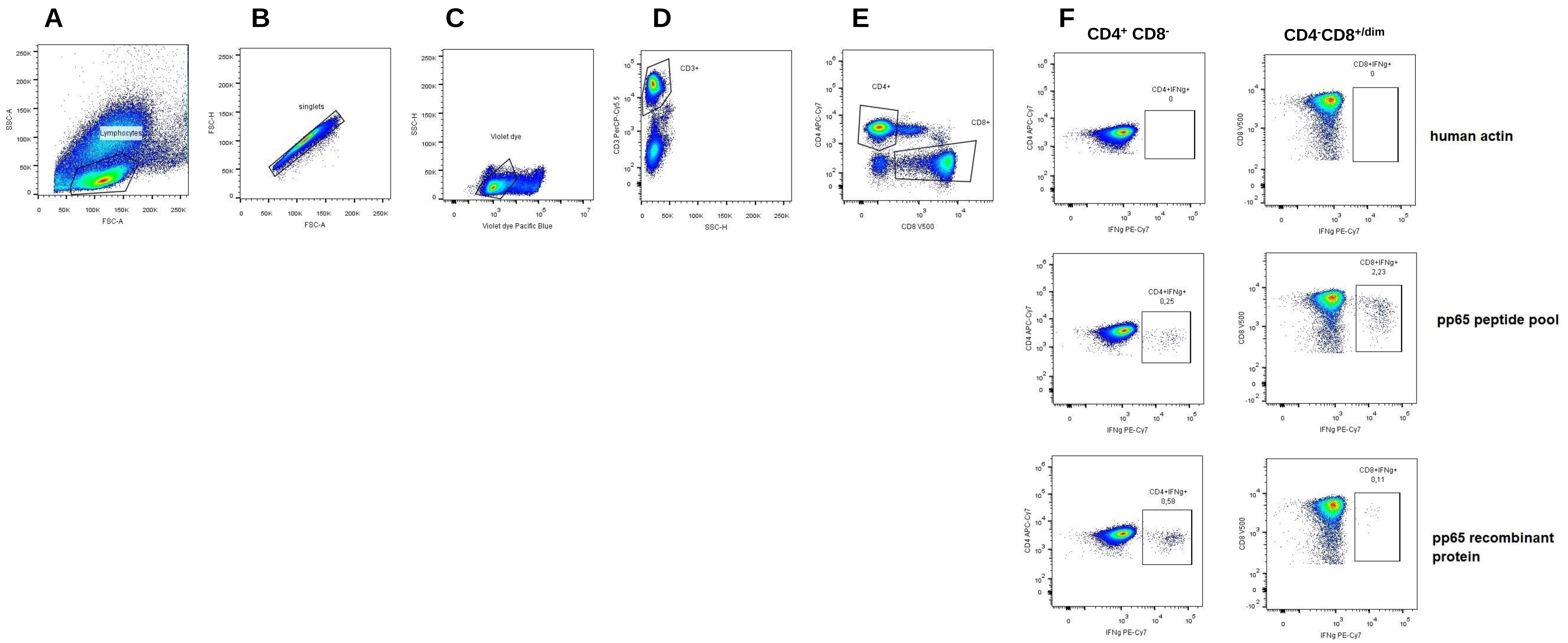

Supplementary Figure 1. Gate strategy used to identify IFN-γ produced by $CD4^+$ and $CD8^+$ T cells. T cells were derived from total live PBMC (A), followed by gating on singlets (B), exclusion of dead cells by a viability stain (C) and gating on $CD3^+$ cells (D). Conventional $CD3^+$ T cells were further divided to $CD4^+$ $CD8^-$ (gate named $CD4^+$) and $CD4^-CD8^{+/dim}$ (gate named $CD8^+$) T cell subset (E). $CD4^+CD8^{+/dim}$ double positive and $CD4^-CD8^-$ double negative T-cell populations were excluded from the following analysis. Representative dot plots showing IFN-γ and CD4 or CD8 expression on $CD4^+$ $CD8^-$ and $CD4^-CD8^{+/dim}$ T cell subsets, respectively, after stimulation (F).
