# Supplementary material for: Performance of new pp65-IGRA for the quantification of HCMV-specific CD4+ T-cell response in healthy subjects and in solid organ transplant recipients
Source: Front Immunol. 2025 May 15;16:1553305. doi: 10.3389/fimmu.2025.1553305 (PMC12119300; doi:10.3389/fimmu.2025.1553305)
Supplement: Supplementary file 3 [file Image2.pdf]

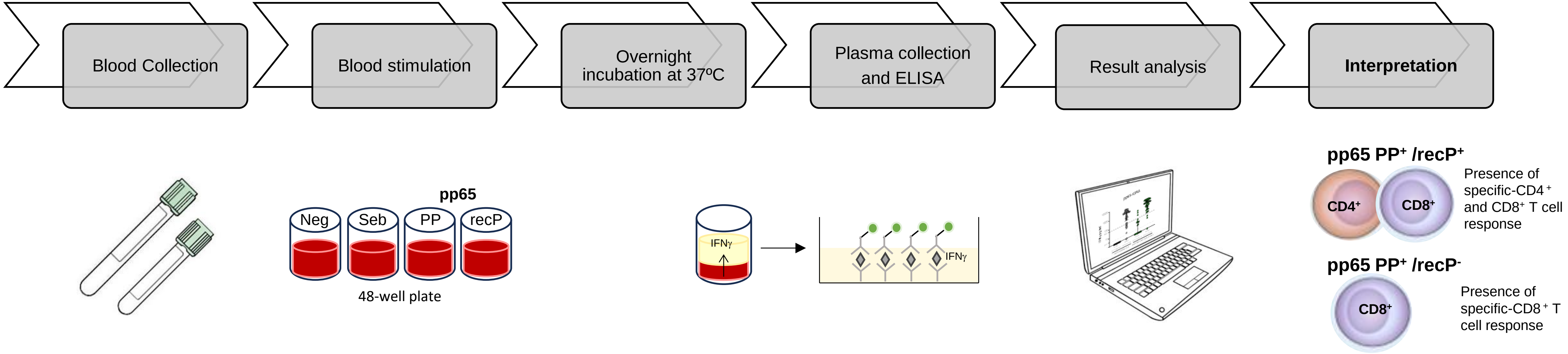

Supplementary Figure 2. The main steps of new pp65-IGRA for the quantification of HCMV specific-T cell response. Abbreviations: pp65 PP, pp65 peptide pool; pp65 recP, pp65 recombinant protein; IFNγ, Interferon–γ; pp65 PP^{+} /$recP^{+}$, presence of a positive response against both the pp65 peptide pool and the pp65 recombinant protein as stimuli; pp65 PP^{+} /$recP^{-}$, presence of a positive response only against the pp65 peptide pool.
